# Impact of the Fukushima Accident on ^3^H and ^14^C Environmental Levels: A Review of Ten Years of Investigation

**DOI:** 10.3390/molecules28062548

**Published:** 2023-03-10

**Authors:** Jakub Kaizer, Ivan Kontuľ, Pavel P. Povinec

**Affiliations:** Faculty of Mathematics, Physics and Informatics, Comenius University, 84248 Bratislava, Slovakia

**Keywords:** Fukushima accident, tritium, radiocarbon, environment, analytical techniques

## Abstract

The investigation of the impact of the Fukushima accident is still going on although more than ten years have passed since the disaster. The main goal of this paper was to summarize the results of tritium and radiocarbon determinations in different environmental samples, possibly connected with the Fukushima Dai-ichi Nuclear Power Plant (FDNPP) accident. A document containing compiled data may serve as a solid basis for further research in the selected fields. To accomplish such effort, we went through dozens of relevant published papers, reporting ^3^H and ^14^C activity concentrations in precipitations, groundwater, seawater, river systems, tree rings, and, in some more extraordinary samples, such as herbaceous plants or debris from the damaged reactor buildings. As the referenced results would not be obtainable without adequate analytical techniques, the most common methods for routine measurement of tritium and radiocarbon concentrations are discussed as well. We believe that the correct identification of the affected environmental compartments could help quantify the released ^3^H and ^14^C activities and track their following fate, which could be especially important for plans to discharge contaminated water from the FDNPP in the upcoming years.

## 1. Introduction

The nuclear accident at the Fukushima Dai-ichi Nuclear Power Plant (FDNPP) was one of the most catastrophic events in the last decade. In March 2011, an exceptionally strong earthquake and the consequent tsunami waves struck the eastern coast of Honshu (Japan), causing severe damage to the critical systems of the FDNPP and eventually leading to a loss of ability to control and cool the shutdown reactors. Despite the enormous effort, hydrogen explosions, which emerged from the reaction of heated steam with nuclear fuel cladding, occurred in three out of four reactor buildings [1]. The total amount of released radionuclides from the FDNPP nuclear core inventories into the atmosphere was estimated to be 9.6 ± 3.4 EBq, comprising mainly short-lived fission products, such as ^133^Xe and ^131,133^I [2]. However, the cores contained many different radionuclides with various half-lives, including tritium (^3^H or T) and radiocarbon (^14^C), which were also emitted as a consequence of the accident, although to a much smaller extent [3,4]. These releases were transported by the vertical movement of air masses and then removed from the atmosphere by dry/wet deposition or other processes, impacting not only areas close to the FDNPP but also regions distant, even regions thousands of kilometers away [5]. In addition to the atmospheric route, radionuclides entered the Pacific Ocean via direct discharges of liquid wastes that were produced by the need to inject water on the melted nuclear reactors. On top of that, heavy rains caused runoffs of contaminated waters from reactor basements and trenches to the coastal region offshore Fukushima [2]. Sporadic releases of increased radioactivity in this area were documented several years after the accident [6]. Horizontal and vertical mixing in the Pacific Ocean allowed for the further spreading of diluted radionuclide concentrations over long distances [7,8,9].

Tritium (*T*_1/2_ = 12.32 yr) is a naturally occurring cosmogenic radionuclide that is produced by interactions of cosmic-ray particles with the nuclei of nitrogen and oxygen in the stratosphere. The steady-state worldwide inventory of natural ^3^H was calculated to be ~2.2 EBq [10], which is less than the nondecayed amount generated during the nuclear weapons testing era in the previous century (~6.0 EBq) [11]. Other sources, such as nuclear accidents, nuclear fuel reprocessing, and operations of nuclear power plants, can be considered minor and may have influence only on the local or regional scale [12,13,14]. Whether it is produced by a natural or anthropogenic process, tritium dominantly enters the environment in the form of water (HTO) or gas (T_2_). Tritiated water molecules can be transferred by means of the hydrological cycle to all compartments. In plants and animals, tritium can then be metabolized into organically bound tritium (OBT) [15,16,17], which is potentially more hazardous than HTO due to its much longer biological half-life. On the other hand, the radiotoxicity of ^3^H is rather low (the maximum energy of emitted beta-particles is only 18.6 keV), which is illustrated by international limits for drinking water, ranging from 100 to more than 70,000 Bq L^−1^, depending on the country [18]. The ingestion dose equivalent for one-year-old children is 4.1 × 10^−11^ and 1.1 × 10^−10^ Sv Bq^−1^ for tritiated water and OBT, respectively [19]. Its unique properties make tritium an excellent tracer for biochemical research [20], groundwater transport measurements [21], and oceanographic studies [22]. Tritium activity is often reported in tritium units (TU), where 1 TU is equivalent to 0.119 Bq L^−1^.

Like tritium, radiocarbon (*T*_1/2_ = 5730 yr) is a cosmogenic radionuclide that is naturally produced by the reaction of neutrons with stratospheric and tropospheric nitrogen and oxygen atoms. After being produced, it is rapidly oxidized to carbon monoxide and consequently to carbon dioxide. Molecules of ^14^CO_2_ are then transported to the lower parts of the atmosphere, absorbed in the hydrosphere, and incorporated into the biomass by photosynthesis, becoming part of the global carbon cycle. The largest reservoir can be found in the oceans where ~6.9 EBq of radiocarbon has been accumulated [23]. While nuclear weapons testing increased the total radiocarbon inventory by ~0.2 EBq, the contribution of other anthropogenic sources can be regarded as almost negligible [10]. Depending on the type of reactor, radiocarbon can be emitted from a nuclear power plant in the form of carbon dioxide or hydrocarbons (^14^C_n_H_n_) [24]. Anthropogenic and natural radiocarbon can be reliably distinguished one from another [25,26,27], which may be important for different studies, exploiting ^14^C as a tracer [28]. From a radiotoxicity point of view, radiocarbon could be relevant because it is contained in all of the organic molecules of living organisms, including DNA, which is susceptible to unrepairable breaks and mutations caused by ionizing radiation [29,30], though the energy of the beta particles it emits is not very high (156 keV). In environmental sciences, the ^14^C concentration is usually given as Δ^14^C (‰) which represents an excess of radiocarbon content relative to a standard [31].

**Figure 1:**
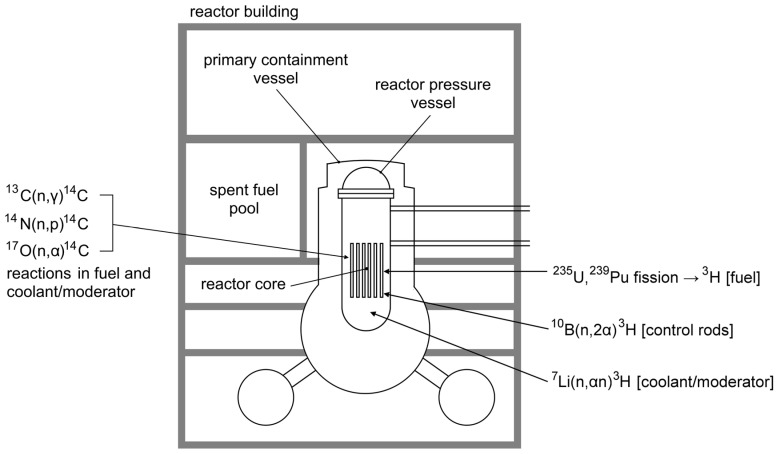
Simplified scheme of a boiling water reactor (BWR) operated at the FDNPP with the typical production pathways of ^3^H and ^14^C.

The FDNPP operated four boiling water reactors (BWR), whose parameters are summarized in Table 1. In the reactors, tritium was generated mainly by ternary fission of ^235^U and ^239^Pu with yields of 0.013% and 0.023%, respectively. Further production options comprised neutron capture on deuterium, reaction on lithium impurities ^7^Li (n, αn) ^3^H present in the water coolant, and reaction on boron ^10^B (n, 2α) ^3^H from the boron carbide control rods [32,33]. According to calculations [34], each reactor contained ~1.0–1.2 PBq of ^3^H, meaning that the ~3.4 PBq could be cumulatively released from three damaged reactors at maximum. Regarding radiocarbon, its production in the FDNPP BWRs by ternary fission of uranium was very low (yield of 0.00016%). Therefore, its formation was dominated by neutron capture on a stable carbon isotope (^13^C) and by reactions ^14^N (n, p) ^14^C and ^17^O (n, α) ^14^C on impurities in the fuel rods, coolant, and moderator [24] (Figure 1). However, the role of impurities in the fuel was probably less significant than expected due to the fact that most of the air composed of nitrogen and oxygen was removed from the FDNPP fuel rods during their manufacture [3]. The total inventory of ^14^C in all FDNPP nuclear reactors on the day of the accident was estimated to be ~1.0–1.6 TBq [35].

The aim of this review is to chronologically summarize the results of tritium and radiocarbon determinations in different environmental samples that were presumably connected with the FDNPP accident. A compiled document containing data obtained through more than ten years of investigation may serve as a solid basis for further research in the selected fields. Although ^3^H and ^14^C are both pure low-energy beta emitters, they are easily incorporated into biological systems, which makes them potentially important for future radiological studies. Moreover, tritium and radiocarbon are suitable for the tracing of various physical, chemical, and geological processes, which could bring interesting answers to some interesting scientific questions. A correct identification of the impacted environmental compartments affected could help quantify the ^3^H and ^14^C activities released from the FDNPP and track their following fate. Since such an effort would not be doable without proper analytical techniques, the most common methods for routine measurement of tritium and radiocarbon concentrations are briefly discussed as well.

## 2. Methods for the Determination of ^3^H and ^14^C

### 2.1. Liquid Scintillation Counting (LSC)

Radionuclides that emit beta particles are readily determined by liquid scintillation counting (LSC). In this technique, the sample containing separated radionuclides of interest is mixed with an organic scintillator cocktail to form a solution that is then counted by a proper detection system, e.g., TriCarb or Quantulus (PerkinElmer, Waltham, MA, USA). The colorlessness and homogeneity of the solution ensure low self-absorption and high counting efficiency [37]. In the case of ^3^H, tritiated water is usually purified by distillation to remove possible interfering radiocontaminants before the LSC sample is prepared and measured, as most of them are significantly less volatile (e.g., ^40^K). The distillation step can be repeated several times if contaminating radionuclides remain in the first distillate. Solid samples are first to be combusted to obtain HTO, which is then measured [38]. Tritium can also be incorporated into benzene molecules that can be synthesized from the original water sample and used as a solvent for a scintillation cocktail [39]. The limit of detection of ~10 mBq for the measurement of ^3^H activity by LSC has been achieved [38], though if the level of tritium in the sample is beyond this value, it can be increased by a factor of 30–100 with the use of electrolytical enrichment [40].

The determination of radiocarbon by LSC is slightly different compared to ^3^H. Unlike tritiated water, it is impossible to directly combine ^14^CO_2_ with a scintillator cocktail. Regardless of whether it was sampled from the air, obtained by combustion of organic material, or by hydrolysis of an inorganic compound, carbon dioxide is first adsorbed into a suitable chemical medium (e.g., NaOH, BaCO_3_, and Carbosorb) or converted to benzene. The former option offers a relatively fast and simple procedure. However, the latter possibility leads to a higher precision of measurement due to a much higher carbon density, as more than 90% of the mass of benzene comes from carbon [41,42,43]. A typical three-step conversion includes the reaction of CO_2_ with molten lithium to produce lithium carbide, the addition of water to obtain acetylene, and finally its catalytic trimerization to benzene, which is stored for a month before LSC measurement to allow radon and its daughter radionuclides to completely decay [44], though the radon problem can be avoided with a modification of the reaction [45]. On top of that, modern detection systems are capable of evaluating radon contamination and applying a correction to the measured radiocarbon activity [46]. The limit of detection for ^14^C determination by LSC has been reported to be ~15 mBq [38,47].

### 2.2. ^3^H-^3^He in-growth Mass Spectrometry

Since tritium has a very low natural abundance (the isotopic ratio ^3^H/^1^H ≈ 10^−18^), it is quite difficult to determine it directly by mass spectrometry. This problem has been solved by the development of ^3^H-^3^He in-growth spectrometry which is based on the measurement of ^3^He produced by the decay of tritium and the subsequent back calculation of its activity [48,49]. The method is so sensitive that the ^3^H/^1^H isotopic ratios can be determined down to ~10^−20^, corresponding to the tritium activity of 1.19 mBq L^−1^ or 0.01 TU [50]. The technique is excellent for the analysis of environmental samples (e.g., seawater) whose tritium concentration is too low for LSC or gas counting, even after electrolytic enrichment. It is the same as for LSC, the water sample needs to be distilled prior to analysis to avoid any possible contamination. After the sample is transferred into a hermetically sealed container, tritium is left to decay for several months to accumulate enough ^3^He. The tritiogenic fraction is purified and spiked with a known amount of the ^4^He standard [51]. The actual measurement of the ^3^He/^4^He isotopic ratio can be conducted with a sector-field noble gas spectrometer [52,53].

### 2.3. Accelerator Mass Spectrometry (AMS)

Although it is possible to use AMS for the determination of ^3^H-labelled molecules [54], the tritium concentrations of typical environmental samples are by about a factor of 100 below its limit of detection. In the case of radiocarbon, the high sensitivity of AMS enables measuring it down to the level of ^14^C/^12^C ≈ 10^−16^ [55], which is sufficient for most types of environmental samples. The technique is based on the separation of the radionuclide of interest from interfering monoatomic and polyatomic isobars present in the accelerated ion beam with respect to their masses and energies. In the case of radiocarbon, it is inserted into the ion source, either in the form of graphite or carbon dioxide (see below), where it is sputtered to produce ^14^C^–^ and other ions. Since ^14^N does not form negative ions, the main potential interferent is not an issue. The generated ions are then analyzed according to their mass and injected into an accelerator in which they gain much higher energies and a positive charge by stripping electrons, leading to the dissociation of molecular ions, and getting, thus, high sensitivity. In the postacceleration part, ^14^C ions are selected by mass–charge analyzers and counted by an end detector.

The preparation of ^14^C samples for AMS is quite complex. The first step is to obtain CO_2_, e.g., by combustion or acidic dissolution of carbonate precipitate, which is then introduced directly into an ion source, or converted to a graphite target. While the former is especially useful for small samples [56,57,58], the latter is utilized in most laboratories. Graphite targets can be prepared by the catalytic reduction of sample CO_2_ with the use of hydrogen and a suitable metal, such as iron, which also serves as the agent for the deposition of synthesized graphite. Both gases and the iron catalyst are enclosed in a reactor, which is heated to 500–600 °C [59,60]. Another option is to completely omit H_2_ and replace it with zinc which can reduce CO_2_ to CO and then CO to graphite when heated to similar temperatures [60]. In recent years, sealed tube graphitization methods have been developed to accommodate the increasing demand for precise ^14^C AMS analyses. As an example of such procedures, sample CO_2_ is cryogenically transferred into a glass tube with iron and titanium hydride which releases hydrogen for reduction after heating in an oven to 500 °C and zinc for reaction with emerging water [61]. The same effect can be achieved with zinc as the sole reducing agent [62,63]. Since graphitization is very sensitive to catalytic poisons (e.g., sulfur and halogens), sample CO_2_ must be purified which is generally carried out by passing the gas through heated copper and silver columns.

## 3. FDNPP Impact on Tritium Environmental Levels

### 3.1. The Concentration of ^3^H in Atmospheric Precipitation and Water Vapor

**Figure 2:**
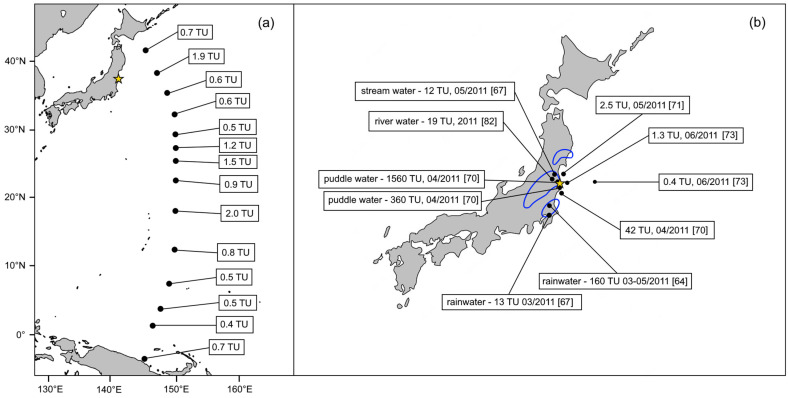
Tritium activity concentrations in various water samples after the FDNPP accident: (**a**) surface seawater values of the western North Pacific Ocean in winter 2012 according to [68], and (**b**) selected 2011 samples from Japan, given with the date of their collection and respective reference [64,67,69,70,71,72]. The yellow star represents the location of the FDNPP. The blue lines show the approximate areas of the significant wet deposition during the second half of March 2011 [2].

Due to the nature of the FDNPP releases, precipitation has been routinely monitored after the nuclear accident, tritium being one of the most interesting radionuclides (Figure 2). The data collected in the southwest direction from the FDNPP site for the period from March to May 2011 showed the highest ^3^H rainwater concentration of ~160 TU in Tsukuba during the first two weeks, leading to an estimation of 1.5 kBq m^−3^ for the source tritium atmospheric activity [64], which corresponds to the ^3^H inventory of 0.6 PBq for a BWR reactor operated for one year [65]. The tritium level decreased steadily in the following days, however, its excess over the background was observed even at the locations 700 km from the damaged nuclear power plant. In the summer of 2011, tritium rainfall activities in Tsukuba were already comparable with pre-Fukushima values [66] which was also true for Chiba, where ^3^H activity in rainwater peaked at 12.7 TU in March 2011 [67].

In addition to precipitation, scientists have been interested in monitoring the atmospheric concentration of HTO. From October 2016 to March 2021, two locations in the vicinity of the FDNPP have been investigated for this purpose [73,74]. The mean HTO concentration (~55 mBq m^−3^) was found to be significantly higher than the background level for the location 1 km south of the FDNPP site while the more distant location did not show any quantifiable impact. The results suggested that the elevated atmospheric HTO concentration could originate in the releases from the FDNPP site, which were transported by winds prevailing at the time of sampling. This means that the FDNPP remains a potential source of atmospheric ^3^H, though the documented levels do not pose an immediate health risk to humans or other living organisms.

### 3.2. The Concentration of ^3^H in the Pacific Ocean and Coastal Seawater

Several studies have been conducted with the aim of evaluating the influence of the FDNPP accident on marine tritium levels. The earliest measurement of coastal seawater in April 2011 revealed a large contamination of 42 TU some 30 km south of the FDNPP site [69]. In the first half of May 2011, the coastal area near the damaged power plant was screened to evaluate potential discharges. From the obtained values, it was concluded that only 0.05 PBq of tritium was directly released from the FDNPP at that time. The surface and subsurface seawater activities of ^3^H varied from 0.08 to 0.29 Bq L^−1^, which was higher than the estimated pre-Fukushima background of 0.07 Bq L^−1^ [70]. The peak concentrations, which were caused by the advection of the coastal current, were found north and south of the FDNPP seaport where ~0.2 Pbq of ^3^H was cumulatively discharged directly into the sea [75]. The extended area offshore Fukushima was investigated again in June 2011. The ^3^H activity concentrations in these samples were determined to be in the range between 0.4 and 1.3 TU, which was a factor of ~3 above the global fallout background [71]. The water column data showed an expected trend of decreasing values with increasing depth; however, the penetration of FDNPP-derived tritium below 100 m was clearly observed for a sampling station very close to the nuclear power plant. The total amount of ^3^H activity released and deposited to the studied area was 0.1–0.5 PBq which was calculated using measures of the ^3^H/^137^C activity ratios and estimated inventory of released and deposited ^137^Cs [76,77]. Furthermore, the coastal waters off the Aomori and Iwate prefectures, located north of Fukushima, were also studied; however, there was no obvious impact of the FDNPP accident on ^3^H surface levels [78].

A situation in the western North Pacific Ocean after the FDNPP accident, which was evidently affected by direct discharges of radioactive water and deposition from the atmosphere [77], was intensively explored in the winter of 2012. The analysis of the radiocesium content (^134,137^Cs) indicated that the ocean waters were influenced by the FDNPP accident, which was also confirmed by tritium measurements. Both surface and vertical profile samples showed increased levels of ^3^H^,^ with the highest value (2.0 TU) found in the area closest to the FDNPP. The tropical region of the western North Pacific Ocean was also affected (up to 1.0 TU on the surface), implying that precipitation was important for the spread of tritium released from the FDNPP. At some locations, tritium penetrated down to 400–500 m or even deeper. Based on surface data and water column inventories, 0.4–1.0 PBq of tritium was deposited in the western North Pacific Ocean [68,79,80].

### 3.3. The Concentration of ^3^H in Freshwater Systems

Terrestrial waters became a target of investigators right after the FDNPP accident. In April 2011, a very high concentration of ^3^H activity of ~184 Bq L^−1^ was determined in a sample of puddle water, collected 1.5 km from the FDNPP site, which is the highest value in any environmental sample documented in the literature. Paddy water was also sampled in the vicinity of the puddle water, yielding a high tritium level of ~68 Bq L^−1^, which means that the ratio of the ^3^H concentration between the puddle and the paddy water was only 0.4 [69]. This would suggest that significantly contaminated rainwater was diluted by mixing with stagnant water in rice paddy fields. The second puddle water sample collected during the same campaign at a more distant location also contained a high concentration of tritium (~42 Bq L^−1^).

The purity of water sources is critical for ecosystems and human lives. Therefore, the need for their monitoring in Japan after the FDNPP events was not surprising at all. Groundwater, stream water, and spring water from two headwater catchments located about 35 km from the FDNPP were analyzed for tritium concentration between May 2011 and June 2012, obtaining mostly background values, with the exception of the May spring water sample (11.7 TU) [67]. In autumn 2012, fifty wells in Fukushima Prefecture were screened that showed a varying ^3^H distribution (1.1–12.9 TU). Although the highest concentrations were determined at locations close to the FDNPP (<25 km) and at shallow depths (<10 m), the deeper and more distant wells appeared not to be affected [81]. With the use of a simple mixing model, precipitation containing tritium released from damaged FDNPP cores was identified as a probable source of increased groundwater values. However, wastewater stored and treated onsite at FDNPP remained a potential risk for groundwater even several years after the accident, as was documented by a recent study. The sump water collected at the boundary in the 2013–2019 period showed an average ^3^H concentration of 20 Bq L^−1^ [82]. Although this is two to four orders of magnitude lower than the ^3^H concentration in well water from the FDNPP site itself, uncontrolled leakages of any amounts of anthropogenic radioactivity into the environment should be avoided.

From 2011 to 2014, a river system in Fukushima Prefecture was extensively studied during base flow conditions and flood events. The peak tritium concentrations of ~2.2 Bq L^−1^, which was well above the natural background, were determined in the case of two small rivers analyzed in the first year of the investigation [72]. The influence of ^3^H released from the FDNPP exponentially decreased to nondistinguishable values over the next three years. The rest of the river system, which was sampled only from 2012, showed similar temporal changes in the tritium content and its unexpectedly good correlation with the ^137^Cs inventory for the same catchment areas. The screening in the same region continued in October 2014 when samples were collected from four estuaries and respective coastal surface seawaters. However, neither rivers nor marine environments were affected by the potential ^3^H contamination [83]. Further monitoring of the Fukushima Prefecture rivers brought the very same result [84].

### 3.4. The Concentration of ^3^H in Biota and Other Samples

The number of organic samples analyzed for the tritium content in connection with the FDNPP accident was quite low, probably due to the complexity of applied treatment protocols compared to, e.g., water. A survey was carried out from March to August 2011 whose main objective was to measure the concentration of free-water tritium (FWT) in herbaceous plant shoots and evergreen tree leaves, collected in the area around the FDNPP site. The impact of FDNPP releases was clearly visible for the first two months, with the highest FWT concentration of 167 Bq L^−1^ found just outside the 20 km evacuation zone [85]. In less than four months, the value at the same location decreased by a factor of ~30, although it remained above the background level. An assumed relation between the FTW concentration and the distance from the FDNPP was observed as well. In the summer of 2012, branches and leaves from cut and living trees and rubble were sampled in the vicinity of the FDNPP reactor buildings and treated for multinuclide radiochemical analysis. The average ^3^H concentrations of the tree and rubble samples were determined to be 0.31 and 0.62 Bq g^−1^, respectively. Tritium activity was fairly uniform in the case of all samples, though there was no obvious correlation between its ^3^H and ^137^Cs content [86]. From 2015 to 2018, the OBT and FWT concentrations were measured in flounders living in the coastal region of Fukushima, reaching only the limits of detection [87].

## 4. FDNPP Impact on Radiocarbon Environmental Levels

### 4.1. The Activity of ^14^C in the North Pacific Ocean and Coastal Seawater

Similar to tritium, radiocarbon was studied in the Fukushima offshore region and western North Pacific Ocean to see if their ^14^C levels were disturbed by the accident. The first samples were collected in June 2011. The average surface Δ^14^C level of −55‰ was obtained for the extended coastal region while the value increased to −20‰ at the depth of 100–200 m for the same area. The maximum contribution of the FDNPP was estimated to be 6% and 9% for surface seawater and the subsurface layer, respectively, though the lack of background data made it difficult to calculate the actual impact [76]. The measured negative Δ^14^C values were somewhat unexpected. However, their origin was explained by the fact that the investigated region was under the influence of the Oyashio current from the North Pacific Ocean. With seasonal variation, the current can create an intrusion that can bring subarctic waters with a much lower radiocarbon concentration southward, reaching even the eastern coast of Japan [88,89].

Recent modeling results suggested that the majority of radiocarbon releases from the FDNPP were transported over the North Pacific Ocean [90] whose surface seawater was first explored several times during the summer months in the 2011–2016 period. However, no elevated radiocarbon concentrations, which could be attributed to the FDNPP impact, were observed in the data set [91]. In the winter of 2012, scientists focused on the western part of the North Pacific Ocean where water columns were also studied, although no clear correlation was found between measured ^14^C activities and the reported radiocesium signal from the FDNPP [68]. The northern negative Δ^14^C values (−40‰), which were under the influence of the Oyashio intrusion described in the previous paragraph, changed to the positive values (68‰) in the south. This trend, together with the uniform mixing of radiocarbon in the surface mixed layers (the depth of 100–200 m), was in good agreement with the previous results [92,93]. The anthropogenic (bomb-produced) ^14^C water column inventories at 35–40°N, which would be noticeably increased in the case of the significant impact of the FDNPP accident, were actually lower. This decline was probably driven by the lowering of bomb-produced radiocarbon to greater depths and its movement along isopycnic layers [80].

### 4.2. The Activity of ^14^C in Tree Rings and Other Samples

Tree rings represent a natural reservoir that can annually accumulate atmospheric radiocarbon, emitted, e.g., from a nuclear power plant (Table 2). The Japanese cedars that were cut down in Iwaki and Okuma some 50 km and 1.5 km away from the FDNPP, respectively, showed a typical temporal exponential decrease of the Δ^14^C values and intensifying Suess effect. In addition, a small ^14^C peak was found for the 2011 Iwaki tree ring, although it could not be identified in the case of the Okuma sample [94,95]. More detailed results were obtained in a study that comprised six additional cedar tree samples, collected in the north and northwestern directions from the damaged nuclear power plant. The average excess ^14^C activity of ~40 Bq kg^−1^ C was measured for two 2011 early wood subring samples from the close vicinity to the FDNPP, followed by a sharp decline to zero in 2012, which would well correspond with the permanent shutdown of the reactors. However, even higher excess ^14^C activity was reported for the same location in 2010 when the reactors were in routine operation [35]. In order to add more values to the dataset, two cypress and cedar trees from the Namie region were analyzed. The results suggested that the maximum impact of the FDNPP accident could be equivalent to the excess ^14^C activity of ~10 Bq kg^−1^ C [96]. Furthermore, a possible southwestern dispersion of radiocarbon from the FDNPP was postulated by the investigation of additional Okuma trees, in which the average late wood Δ^14^C value reached ~260‰ over the background. The higher contamination of the late wood rather than the early wood was explained by potential post-accidental releases [97]. The most recent study confirmed that no influence on the radiocarbon level in trees or plants can be anticipated beyond the 30 km border from the FDNPP site [90].

Except for already discussed seawater and tree rings, there have been only a few other radiocarbon samples documented in the literature which could be connected to the FDNPP. In November 2011, a leaf litter sample was collected in Kawamata in northeast Japan and divided into two distinct fractions. Despite their quite high content of radiocesium, neither of the fractions yielded an increased ^14^C concentration which was in concordance with the atmospheric level [98]. The radiocarbon screening of the surrounding of the FDNPP reactor buildings, which was conducted in the summer of 2012, has brought some interesting results. While the ^14^C activity in the unit one rubble sample, together with tree branches and leaves, was measured to be below the limit of detection, its value for most of the rubble samples of units three and four was in the range of 0.13–2.7 Bq g^−1^ [86]. This would suggest that no large amount of radiocarbon was dispersed away from the reactor buildings.

## 5. Summary and Perspectives

Even though more than ten years have passed since the disaster, the investigation of the FDNPP accident’s impact on the environmental tritium and radiocarbon levels is still ongoing. In contrast to radiocesium, tritium and radiocarbon remain fairly understudied. There are some crucial FDNPP-connected aspects that we do not completely understand, e.g., total releases of radiocarbon and its deposition into the marine environment, inhomogeneous distribution of the released radionuclides of interest in different samples and magnitude of their intake by population, their incorporation on the molecular level and its potentially harmful effects, etc. This discrepancy between the radionuclides is mainly caused by the present background, which is almost negligible in the case of radio-cesium, while the nuclear weapon test legacy and the natural production of ^3^H and ^14^C are quite significant and can make a fresh anthropogenic signal difficult to detect. The problem has become less critical with the option to apply modern analytical methods. However, even their great capabilities might be insufficient in some cases. Further development of ultrasensitive techniques could be essential in order to push the limits of the research connected with the long-lived radionuclides released from the FDNPP.

The government of Japan has recently agreed to discharge into the Pacific Ocean radioactive wastewater which has been accumulated at the FDNPP site. The water still contains significant amounts of ^3^H and ^14^C which cannot be removed in a reasonable way by any physical or chemical process. The current estimations suggest that the total activity of tritium stored in tanks is ~1 PBq while the value is by four to five orders of magnitude lower in the case of radiocarbon [99]. Although the amount of ^3^H is higher than the calculated activity already released from the FDNPP, a plan to discharge the wastewater over a few decades should minimize its impact on the environment. However, there are several uncertainties that can strongly affect the actual prediction [100], though with more details of the discharge plan revealed, scientists can improve their transport models and obtain more realistic outcomes on the fate of discharged radionuclides [101,102]. The obtained modeled data should then be compared with the values from sample analyses. Therefore, the monitoring of tritium and radiocarbon activity in the Pacific Ocean will be important during and after the release campaign.

Future radioecological and environmental studies will require additional relevant post-FDNPP accident samples, which will help to gather more relevant ^3^H and ^14^C data. For the former, such an effort could be complicated due to its shorter half-life. However, the latter does not have this disadvantage and inorganic and organic molecules labeled with the FDNPP-derived radiocarbon will be part of the environment for millennia. As documented in several papers, tree rings have proven to be able to mirror the ^14^C concentration in the atmosphere around the FDNPP. Corals or other appropriate organisms could play a similar role in the Pacific Ocean. However, the topic of organically bound tritium (OBT) has been barely explored in the literature regarding the FDNPP accident and deserves more attention. In general, the increased number of ^3^H and ^14^C determinations will lead to a better evaluation of the released radionuclide inventories and transport pathways. This knowledge would improve the ability to exploit radionuclides as tracers in various physical and chemical processes in the atmosphere–biosphere–hydrosphere (ocean) ecosystems.

## Figures and Tables

**Table 1:** Basic parameters of the FDNPP boiling water reactors [36].

Unit Number	1	2	3	4
Reactor type	BWR-3	BWR-4	BWR-4	BWR-4
Type of fuel	LEU	LEU	MOX	LEU
Moderator	Light water			
Coolant	Light water			
Start of operation	March 1971	July 1974	March 1976	October 1978
Thermal output (MW_t_)	1380	2381	2381	2381
Electric output (MW_e_)	460	784	784	784
No. of fuel assemblies	400	548	548	548
Status prior to the accident	Operational	Operational	Operational	Shutdown
Estimated total core inventory (EBq)	14.4	24.4	24.5	-

**Table 2:** Average measured radiocarbon activity in Japanese tree rings from 2011.

Location	Direction from the FDNPP	Distance from the FDNPP	Δ^14^C (‰)	^14^C activity (Bq kg^−1^ C)	Reference
Okuma	SW	1 km	40.3	233.4	[95]
			37.5	232.8	[97]
			256.5	284.0	[97]
			266.8	285.4	[97]
Futaba	NW	2.5 km	152.4	258.2	[35]
Namie	N	8 km	23.3	230.7	[35]
Tomioka	S	9 km	48.6	236.3	[97]
			73.5	241.9	[97]
Takase	NW	11 km	65.7	240.0	[35]
Ogaki	NW	14 km	57.2	237.8	[35]
Shimotsushima	NW	32 km	38.4	232.7	[35]
Yamakiya	NW	38 km	28.9	232.9	[35]
Iwaki	SW	50 km	32.9	232.2	[35]
Koriyama	W	60 km	36.3	233.1	[90]

## Data Availability

Not applicable.
